# Characteristics of patients with difficult-to-treat rheumatoid arthritis: a descriptive retrospective cohort study

**DOI:** 10.1186/s42358-024-00396-6

**Published:** 2024-08-06

**Authors:** Wen Qi, Antoine Robert, Narcisse Singbo, Lucie Ratelle, Paul R. Fortin, Louis Bessette, Jacques P. Brown, Laëtitia Michou

**Affiliations:** 1 Division of Rheumatology, CHU de Quebec-Université Laval, Quebec City, Canada; 2 Department of Medicine, Université Laval, Quebec City, Canada; 3 Research Center of the CHU de Québec-Université Laval, Quebec City, Canada; 4 Osteoporosis and Rheumatology Center of Quebec, Quebec City, Canada; 5 Centre ARThrite– UL, Quebec City, Canada; 6 Rhumatologie-R4774, CHU de Québec-Université Laval, 2705 boulevard Laurier, Quebec City, QC G1V 4G2 Canada

**Keywords:** Rheumatoid arthritis, Fibromyalgia, Antirheumatic agents, JAK inhibitors

## Abstract

**Background:**

In 2021, an EULAR task force published a definition of difficult-to-treat rheumatoid arthritis (D2T RA). Our current knowledge of D2T RA with the EULAR definition is based on European and Asian cohorts, and no North American cohort has yet to be published. The aim of this study was to compare D2T RA patients to non-D2T RA who are good responders to advanced therapy, and to describe their evolution in an university health center patient cohort.

**Methods:**

This is a retrospective single centre study of the medical records of all adults with RA on at least one biologic or target synthetic DMARD (b/tsDMARD). D2T RA group was defined according to the EULAR definition of D2T RA. The non-D2T RA group was defined as a b/tsDMARD good responder who had low-disease activity or remission for at least one year on 1 or 2 b/tsDMARD mechanism of action. We compared the patients’ comorbidities, and history of b/tsDMARD use. Descriptive statistics and proportions were calculated. Kaplan-Meier analysis with log-rank test was used to estimate and compare median survival.

**Results:**

Among the 417 patients, 101 (24%) were D2T RA and 316 (76%) were non-D2T RA. D2T RA group was slightly younger (63 ± 9 years versus 65 ± 12 years, *p* = 0.045), more likely to have concomitant non-inflammatory pain (28% versus 8%, *p* < 0.0001) and to discontinue at least one b/tsDMARD due to intolerance (39% versus 10%, *p* < 0.0001). In the D2T RA group, JAK inhibitors were associated with longer drug continuation when used as the third b/tsDMARD. Fewer patients were using corticosteroid at their most recent follow-up in this Canadian cohort compared to others (16% versus from 29 to 74%).

**Conclusion:**

Concomitant non-inflammatory pain was more prevalent in D2T RA patients compared to b/tsDMARD good responder non-D2T RA patients. Steroid-sparing strategies is possible even in D2T RA patients. Future prospective research may compare JAK inhibitors with other mechanisms of action in D2T RA.

**Supplementary Information:**

The online version contains supplementary material available at 10.1186/s42358-024-00396-6.

## Introduction

Rheumatoid arthritis (RA) is a chronic systemic inflammatory disease that affects 0.5–1% of the world population [1, 2]. The treat-to-target approach in RA, in which treatment is regularly adapted until a desired low disease activity state is achieved, has been greatly influenced by the introduction of new biologic and target synthetic disease-modifying antirheumatic drugs (b/tsDMARDs) [3]. However, 8–20% of patients fail to reach treat-to-target objectives despite recent therapeutic improvements [4, 5]. The persistence of high disease activity leads to a greater burden of disease [6] and an estimated 18,000 euros (equivalent to 20,195 US dollar) per year in average cost increase [7]. Some cohort studies [8–10] explored factors predicting failure to multiple different b/tsDMARDs, but they lacked a common definition of treatment-refractory RA. In 2021, a EULAR Task Force [11–13] defined “difficult-to-treat RA” (D2T RA) [14] and they later published points to consider in the management of patients with D2T RA [15].

Understanding the determinants of D2T RA is key to tailoring its management. The current hypotheses for D2T RA include: (1) Presence of other causes of pain (e.g.: chronic pain syndromes, osteoarthritis, damage) [6, 16, 17], (2) Drug tolerance issues related to comorbidities or more frequent adverse events (e.g.: lung disease, infections) [18–20], (3) Socioeconomic challenges (e.g.: drug cost, poor coping skills, unrealistic patient expectations) [21] and (4) True multidrug resistance. Our current knowledge of D2T RA with the EULAR definition is based on European and Asian cohorts, and no North American cohort has yet to be published. Rheumatology practice varies between countries and continents due to differences in guidelines [22, 23] and prescription habits [24, 25].

The primary objective of this study was to identify characteristics of patients with D2T RA (b/tsDMARD poor responders) compared to b/tsDMARD good responders, and to describe their evolution in an university health center patient cohort. The secondary objective was to describe the type of b/tsDMARD associated with the longest duration on treatment after a patient was classified as D2T RA.

## Methods

### Study design and patient selection

This is a retrospective single centre cohort study at our university hospital. This study was approved by our institution Ethics Committee (IRB number 2020–5081). After obtaining authorization from the Director of Professional Services of our hospital, we reviewed the electronic medical records of all adults with RA that met the ACR/EULAR 2010 classification criteria [26] and had been seen in our outpatient clinic. All participants had to have been treated with at least one biologic or target synthetic DMARD (b/tsDMARD) to be included. Patients who were never treated with a b/tsDMARD were excluded, because the aim of this study was compare D2T RA patients (poor responders to b/tsDMARDs) to b/tsDMARD good responders. Patients were excluded from the analysis if (1) they only have been treated with conventional synthetic DMARDs (csDMARDs), (2) they had a concomitant rheumatic disease that could cause chronic small joint polyarthritis (e.g.: systemic lupus erythematosus and RA overlap, psoriatic arthritis, inflammatory bowel disease), or (3) their file contained limited information (e.g.: patients who are not seen at least annually at our outpatient clinic. To better study true failure of a mechanism of action, a b/tsDMARD was excluded from our analysis if it was discontinued due to intolerance.

### Definitions and outcome variables

To apply the EULAR D2T RA definition retrospectively, patients must have failed ≥ 2 b/tsDMARDs with different mechanisms of action. The decision of introducing or changing a b/tsDMARD was made by the patient’s rheumatologist, in compliance with ACR and EULAR RA guidelines. To ensure that all patients in the D2T RA group had signs of active or progressive disease to fulfill the second EULAR D2T RA criteria, we documented the swollen joint count [28], tender joint count [28], inflammatory markers (erythrocyte sedimentation rate/C-reactive protein), the presence of erosions on X-rays and the health assessment questionnaire (HAQ) score at their most recent visit and at any time a b/tsDMARD was discontinued. A patient entered the D2T RA group after failing their second b/tsDMARD mechanism of action. The non-D2T RA group was defined as low disease activity or remission for at least one year on 1 or 2 b/tsDMARD mechanisms of action. Both groups were mutually exclusive.

Data collection was done by three investigators between April 2020 and September 2021: two core internal medicine residents in their second or third year of training (WQ, AR), and one research assistant (LR). All files reviewed by the research assistant (*n* = 17/420 files) were subsequently verified by a resident (WQ). The patient’s demographic information and comorbidities were collected from the patient’s hospitalization summaries and medical records (all rheumatology records and consultation records in other medical specialties) since 2015. Based on a predetermined standardized data collection form, we collected information on patient’s demographics (age at their most recent rheumatology follow-up, biological sex) and their medical/psychological comorbidities: hypertension, diabetes, dyslipidemia, chronic kidney failure, liver disease, concomitant non-inflammatory pain, anxiety disorders, and mood disorders. The presence and titer of rheumatoid factor (RF) and anti-citrullinated protein antibody (anti-CCP) were recorded from the earliest information available. ESR/CRP were documented categorically (elevated or normal, based on the laboratory’s reference values) and numerically. Erosions on X-rays and rheumatoid nodules were document categorically (present or absent). Concomitant of use of conventional synthetic DMARDs (csDMARDs), non-steroidal anti-inflammatory drugs (NSAIDs) and corticosteroids were documented categorically (presence or absence). For both groups, we documented the chronological order of use of b/tsDMARDs according to their mechanism of action.

Concomitant non-inflammatory pain was defined as the diagnosis of fibromyalgia, complex general pain syndrome or chronic pain syndrome by the patient’s rheumatologist. This outcome was considered positive only if the disease activity was well controlled according to the rheumatologist and the patient still had symptoms causing a reduction in their quality of life, at any given time since 2015.

When a b/tsDMARD was discontinued for an intolerance, the type of intolerance was classified as follows: an allergic reaction, management of comorbidities, and side effects. An allergic reaction was defined as urticaria, angioedema, hypotension, or bronchospasm. The management of comorbidities was defined as (1) the drug became contra-indicated because of another health condition (e.g.: cancer and chemotherapy), or (2) the exacerbation of a preexisting comorbidity due to the drug (e.g.: lung infections in a patient with chronic lung disease). Side effects were defined as (1) a non-allergic effect attributed to the medicament, or (2) a severe infection in a patient without an identified predisposing comorbidity (ex: immunodeficiency, chronic lung disorder). Drug access issues or non-adherence were also recorded. Drug access issues were defined as the patient could no longer have access to the drug due to systemic factors (e.g.: insurer refused to pay for the drug, medication withdrawn from the Canadian market). Non-adherence was defined as the clinical diagnosis of non-adherence by the rheumatologist.

### Statistical analysis

The patient’s demographics and characteristics were reported using descriptive statistics (mean and standard deviation or median with interquartile ranges) for continuous variables, and relative frequencies (%) for categorical variables. Univariate logistic regression models with odds ratios (OR) estimations were used to assess the characteristics (categorical and continuous) which were associated and could predict the D2T RA status. Characteristics chosen as candidate predictors were selected based on prior (clinical and literature) knowledge of their association with the D2T RA status. Kaplan-Meier analysis with log-rank test was used to estimate and compare median survival between the types of b/tsDMARD. The missing values for some variables and the non-collection of some possible confounders were limitations to perform multivariable analysis, thus we chose Kaplan-Meier bivariate analysis instead of a multivariate Cox regression for survival analysis. The analyses were performed (SM) using SAS 9.4 software. Missing data were managed as missing. A P value < 0.05 was considered statistically significant.

## Results

### Patient characteristics

**Fig. 1:**
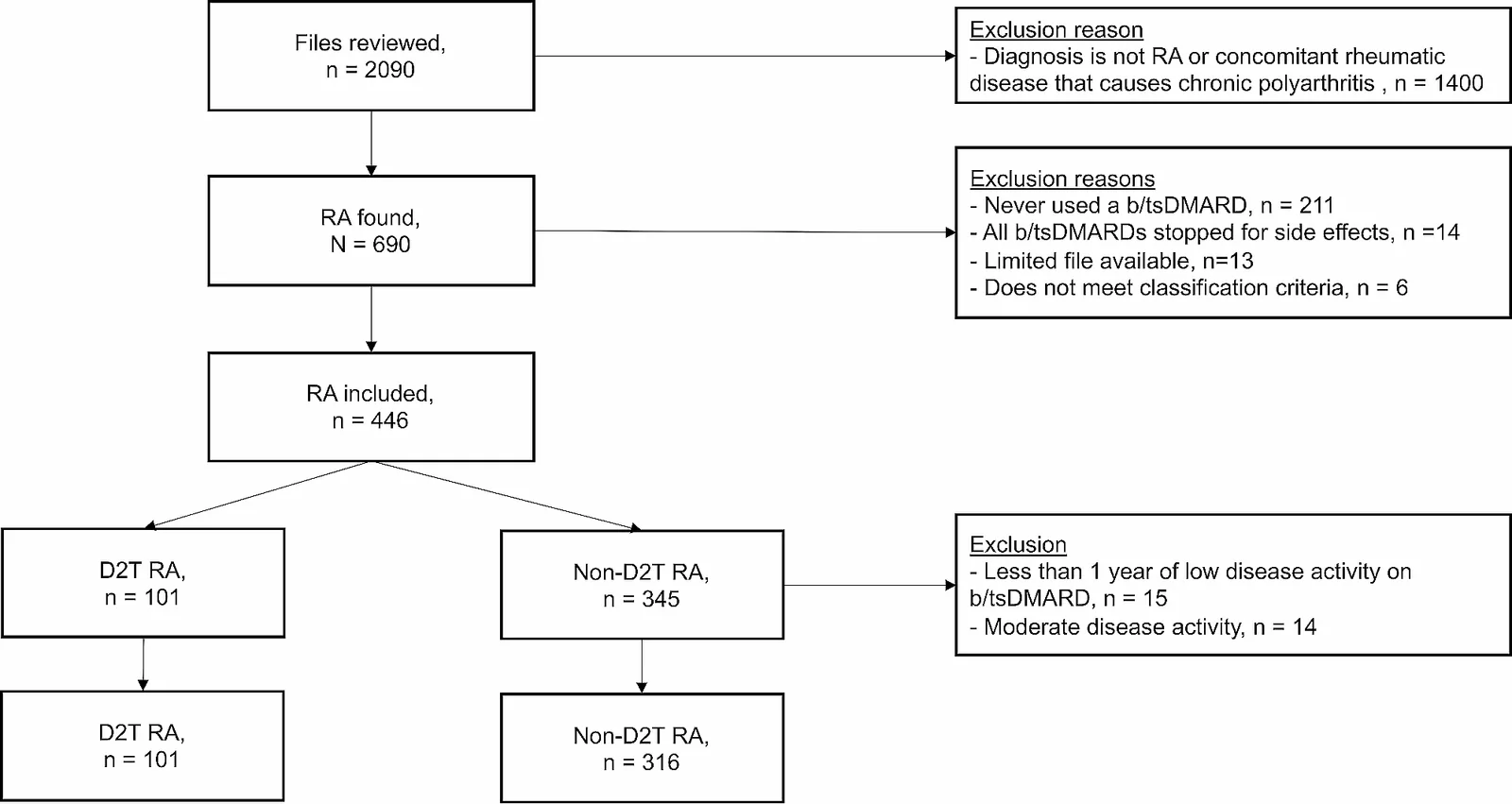
Patient recruitment. *Note RA* rheumatoid arthritis, *D2T RA* difficult-to-treat rheumatoid arthritis, *non-D2T RA* non-difficult-to treat rheumatoid arthritis, *b/tsDMARD* biologic or target synthetic disease-modifying antirheumatic drug

After reviewing records from 2090 patients with a chronic inflammatory arthritis, we enrolled 417 RA patients on b/tsDMARDs (Fig. 1): 101 (24%) were D2T RA and 316 (76%) were non-D2T RA (Table 1).

**Table 1:** Patient and rheumatoid arthritis characteristics

	D2T RA (*n* = 101)	non-D2T RA (*n* = 316)	Odds ratio (95% CI)	*P* value
**Demographic information**				
Female, n (%)	81/101 (80)	238/316 (75)	0.75 (0.43–1.31)	0.3149
Age at most recent follow-up, mean (S.D.) years	63 (9)	65 (12)	0.98 (0.96–1.00)	0.0455
Age at RA diagnosis, mean (S.D.) years	49 (13)	51 (15)	0.99 (0.98–1.01)	0.2348
Duration of disease since RA diagnosis, mean (S.D.) years	16 (12)	18 (14)	0.99 (0.98–1.01)	0.5505
Time of follow-up since inclusion in each study group, mean (S.D.) years	5 (3)	9 (5)	–	
Duration before meeting D2T criteria, median (Q1–Q3) months	37 (20–69)	–	–	
Duration before the first b/tsDMARD, median (Q1–Q3) years	2 (1–8)	3 (1–11)		0.0134
Number of b/tsDMARDs, median (Q1–Q3)	4 (3–6)	1 (1–2)		
Number of b/tsDMARDs mechanisms, median (Q1–Q3)	3 (3–4)	1 (1–1)		
**Comorbidities**				
Hypertension, n (%)	42/101 (42)	125/312 (40)	1.06 (0.68–1.68)	0.7868
Diabetes, n (%)	14/101 (14)	44/312 (14)	0.98 (0.51–1.87)	0.9158
Dyslipidemia, n (%)	34/101 (34)	83/312 (27)	1.40 (0.86–2.27)	0.1721
Chronic kidney failure, n (%)	5/101 (5)	19/297 (6)	0.76 (0.28–2.10)	0.5988
Cirrhosis, n (%)	1/99 (1)	3/297 (1)		
Concomitant non-inflammatory pain, n (%)	26/92 (28)	24/292 (8)	4.40 (2.37–8.15)	< 0.0001
Anxiety disorder probable, n (%)	8/87 (9)	18/282 (6)	1.49 (0.62–3.55)	0.3725
Mood disorder probable, n (%)	12/92 (13)	25/293 (9)	1.61 (0.77–3.35)	0.2032
**Smoking status on most recent treatment**				
Active smoker, n (%)	18/87 (21)	45/273 (17)		0.3369
Past smoker, n (%)	29/87 (33)	78/273 (28)		
Never smoked, n (%)	40/87 (46)	150/273 (55)		
**Disease characteristic at RA diagnosis**				
Seropositive rheumatoid arthritis, n (%)	53/101 (53)	198/316 (63)	1.52 (0.97–2.39)	0.0697
Rheumatoid factor positive, n (%)	50/99 (51)	169/305 (55)	0.82 (0.52–1.29)	0.3951
RF quantity, mean (95% CI)	214 (101–325)	237 (159–315)	1.00 (1.00–1.00)	0.7207
Anti-CCP positive, n (%)	38/90 (42)	136/265 (51)	0.69 (0.43–1.12)	0.1367
CCP quantity, mean (95% CI)	95 (68–121)	151 (128–174)	1.00 (0.99-1.00)	0.0035
Elevated ESR, n (%)	32/85 (38)	125/243 (51)	0.57 (0.34–0.95)	0.0294
Elevated CRP, n (%)	24/64 (38)	89/166 (54)	0.52 (0.29–0.94)	0.0297
**Medication with most recent b/tsDMARD**				
Still treated with b/tsDMARD, n (%)	97/101 (96)	316/316 (100)		
Use c/sDMARD with b/tsDMARD, n (%)	68/101 (67)	248/316 (79)		0.0227
Use corticosteroids with b/tsDMARD, n (%)	16/101 (16)	15/316 (5)		0.0002
Use NSAID with b/tsDMARD, n (%)	12/101 (27)	36/316 (11)		0.8934
**Disease activity on most recent b/tsDMARD**				
ESR, mean (S.D.)	12 (14)	9 (10)		0.1755
CRP, mean (S.D.)	5 (8)	3 (5)		0.4301
Swollen joint count (28), mean (S.D.)	1 (2)	0 (0)		< 0.0001
Tender joint count (28), mean (S.D.)	3 (4)	0 (1)		< 0.0001
HAQ, mean (S.D.)	0.9 (0.6)	0.3 (0.4)		< 0.0001
Erosion on X-Ray, n (%)	25/82 (31)	82/198 (41)	0.62 (0.36–1.07)	0.0882
Rheumatoid nodule, n (%)	13/57 (23)	32/127 (25)	0.88 (0.42–1.83)	0.7274
**b/tsDMARDs excluded**				
Patients with at least one excluded b/tsDMARD, n (%)	39/101 (39)	32/316 (10)	5.58 (3.25–9.60)	< 0.0001
Number of excluded b/tsDMARD per patient with at least one excluded b/tsDMARD, mean (S.D.)	1.5 (0.7)	1.1 (0.4)		0.006
**Reasons for excluding a b/tsDMARD from analysis**				
Allergic reaction, n (%)	15/59 (26)	8/36 (22)		0.909
For the management of comorbidities, n (%)	9/59 (15)	3/36 (8)		
Side effects, n (%)	32/59 (54)	23/36 (64)		
Non-adherence, n (%)	1/59 (2)	0		
Drug access problem, n (%)	2/59 (3)	2/36 (6)		

*RA* rheumatoid arthritis, *D2T RA* difficult-to-treat rheumatoid arthritis, *non-D2T RA* non-difficult-to-treat rheumatoid arthritis, *b/tsDMARD* biologic or target synthetic disease-modifying antirheumatic drug, *csDMARD* conventional synthetic disease-modifying antirheumatic drug, *RF* rheumatoid factor, *anti-CCP* anti-cyclic citrullinated peptide, *ESR* erythrocyte sedimentation rate, *CRP* C-reactive protein, *NSAID* nonsteroidal anti-inflammatory drug, *ESR* erythrocyte sedimentation rate, *CRP* C-reactive protein

Patients were predominantly women (D2T 80% versus non-D2T 75%, *p* = 0.31). The mean age at inclusion was slightly younger in the D2T RA group (D2T RA 63 ± 9 years, and non-D2T RA 65 ± 12 years, *p* = 0.04). The mean duration of disease since RA diagnosis was similar (D2T RA 16 ± 12 years and non-D2T RA 18 ± 14 years, *p* = 0.25). The time of follow-up since inclusion in the D2T RA group was 5 ± 3 years, and it was 9 ± 5 years in the non-D2T RA group. D2T RA patients had a median disease duration of 37 months (IQR = 20–69 months) before meeting the EULAR 2021 D2T RA definition. They received a median of 4 b/tsDMARDs (IQR = 3–6) with a median of 3 different mechanisms of action (IQR = 3–4). The non-D2T RA group received a median of 1 b/tsDMARD (IQR = 1–2) with a median of 1 mechanism of action (IQR = 1–1).

At RA diagnosis, D2T RA patients were less likely to have an elevated inflammatory marker as defined by ESR (D2T RA 38% versus non-D2T RA 51%, *p* = 0.03) and CRP (D2T RA 38% versus non-D2T RA 54%, *p* = 0.03). Concomitant non-inflammatory pain was more prevalent in our D2T RA group (D2T RA 28% versus non-D2T RA 8%, *p* < 0.0001) (Table 1). D2T RA patients were also more likely to discontinue at least one b/tsDMARD due to intolerance (D2T RA 39% versus non-D2T RA 10%, *p* < 0.0001). However, there were no differences between the two groups in the types of intolerance: side effects, allergic reaction, or due to comorbidities (Table 1).

### How b/tsDMARD were used

**Fig. 2:**
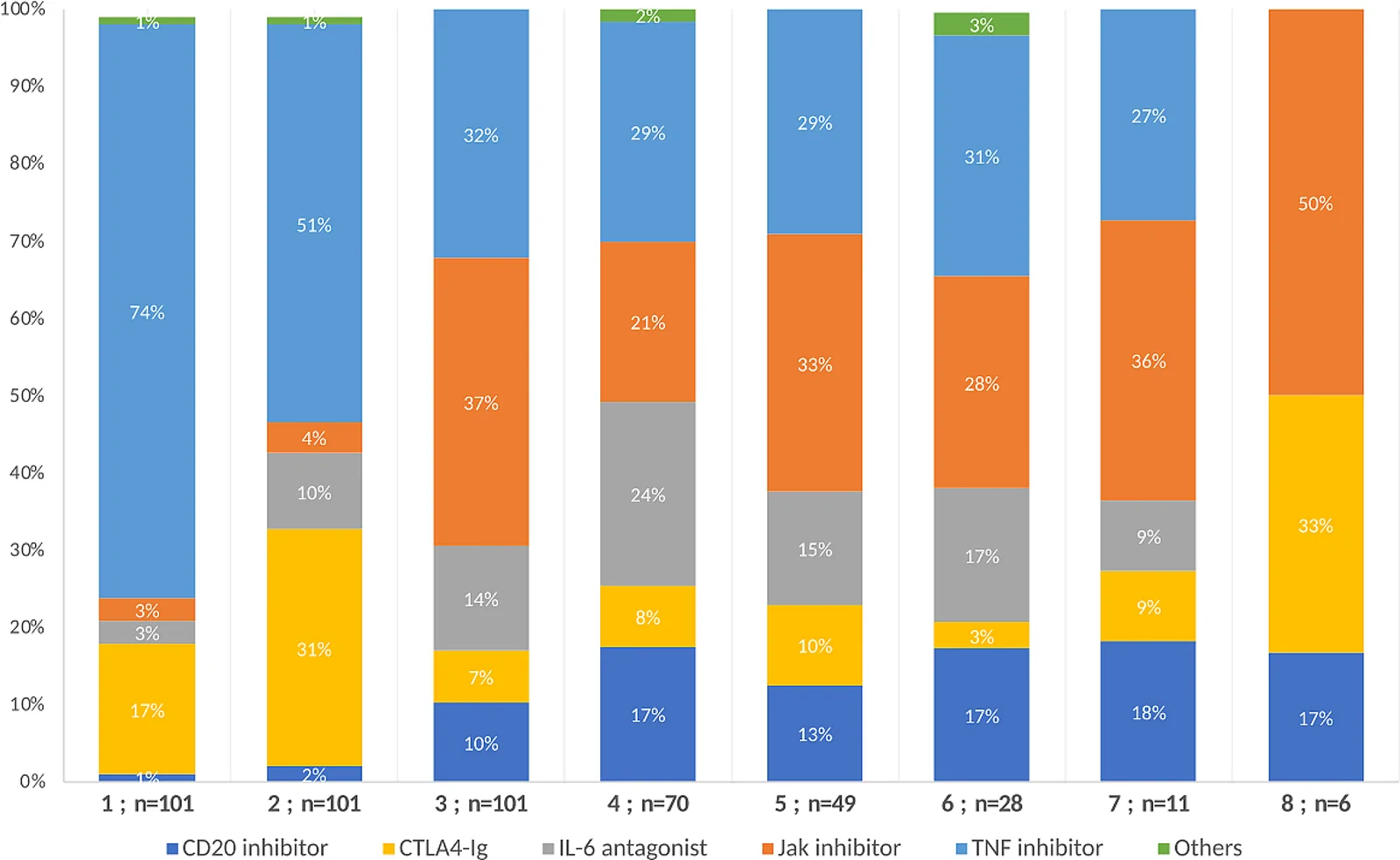
Chronological order of use of b/tsDMARDs according to their mechanism of action in D2T RA. *Note* This figure contains information on the D2T RA group only. *TNF inhibitor* tumor necrosis factor inhibitor (adalimumab, etanercept, golimumab; certolizumab, infliximab), *IL-6 antagonist* interleukin-6 antagonist (tocilizumab, sarilumab), CD20 inhibitor (rituximab), *JAK inhibitor* janus kinase inhibitor (tofacitinib, baricitinib, upadacitinib), *CTLA-4 Immunoglobulin* cytotoxic T lymphocyte-associated antigen-4-immunoglobulin (abatacept), Others (includes Il-1 inhibitor, clinical trial b/tsDMARDs)

In the D2T RA group, the first b/tsDMARD mechanism of action patients received in their pre-D2T RA period were TNF inhibitors (75/101, 74%) and CTLA-4-Ig (17/101, 17%) (Fig. 2), with a median duration of treatment of 11 months (IQR = 7–33) and 8 months (IQR = 6–11), respectively (Supplementary Fig. S1). For their second b/tsDMARD in the pre-D2T RA period, the most frequent mechanism of action was still a TNF inhibitor (52/101, 51%) and CTLA-4-Ig (31/101, 31%), with a median duration of treatment of 7 months (IQR = 5–17) and 12 months (IQR = 5–21 months), respectively (Supplementary Fig. S2).

**Fig. 3:**
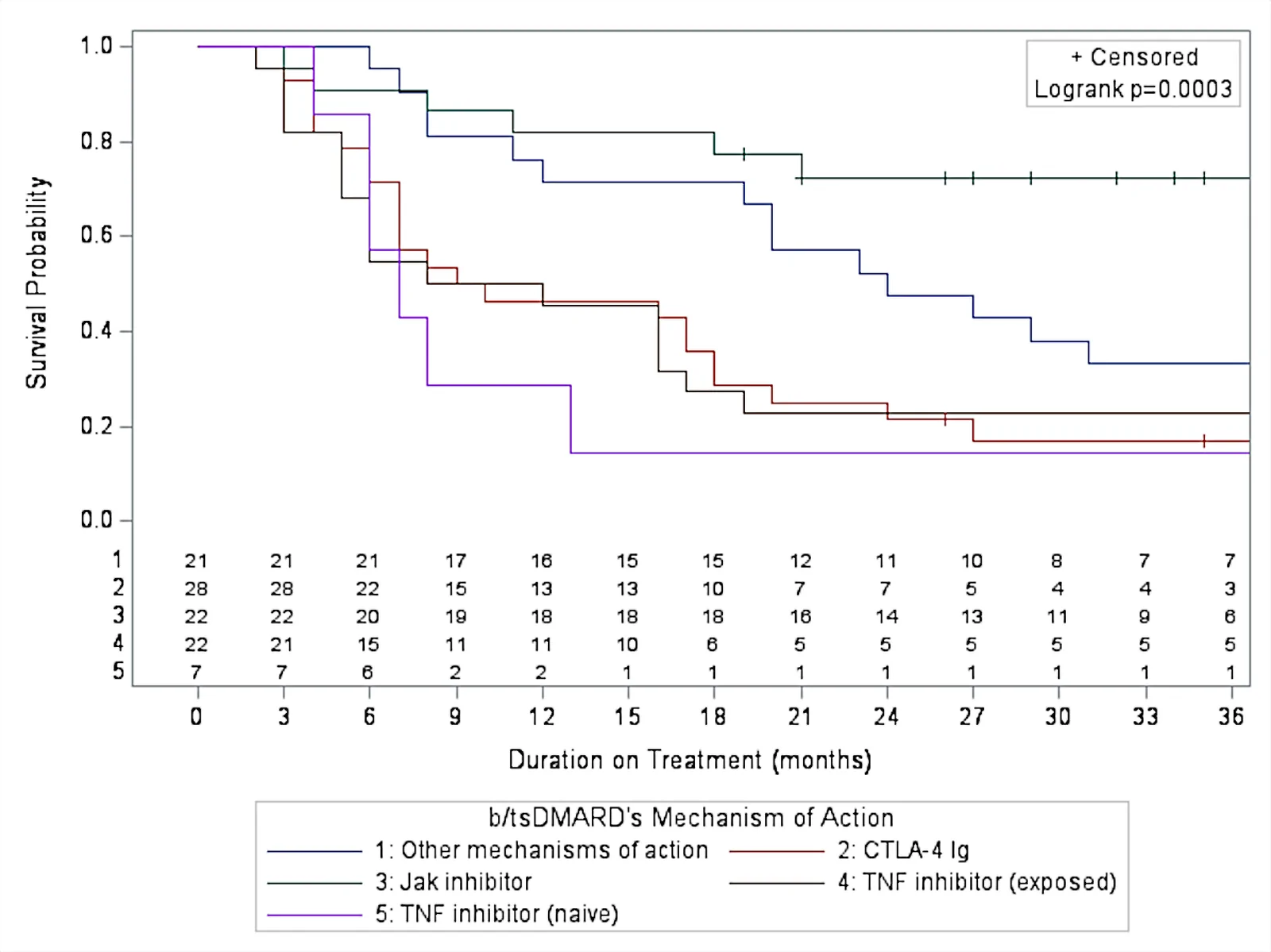
Continuation probability of the third b/tsDMARD up to 36 Months in D2T RA patients. *Note* This information is for follow-up after D2T criteria were fulfilled. *TNF inhibitor* tumor necrosis factor inhibitor (adalimumab, etanercept, golimumab; certolizumab, infliximab); TNF inhibitor (naïve) describes patients who are using TNF inhibitor for the first time; TNF inhibitor (exposed) describes patients who have previously used a TNF inhibitor in the past, *JAK inhibitor* janus kinase inhibitor (tofacitinib, baricitinib, upadacitinib), *CTLA-4 Ig* cytotoxic T lymphocyte-associated antigen-4-immunoglobulin (abatacept); Other mechanisms of action: *IL-6 antagonist* interleukin-6 antagonist (tocilizumab, sarilumab), CD20 inhibitor (rituximab)

The majority of D2T RA patients failed two mechanisms of action after using 2 b/tsDMARDs (*n* = 58/101, 58%) (Supplementary Table S1). D2T RA patients had signs of active disease when a b/tsDMARD was discontinued (Supplementary Table S2). For patients who met the D2T RA criteria and used their third b/tsDMARD, the most frequently used mechanism of action was a JAK inhibitor (22/59, 37%) followed by a TNF inhibitor (19/59, 32%) (Fig. 3). For the fourth b/tsDMARD, there was a more diverse mixture of mechanisms of action: TNF inhibitors (18/63, 29%), IL-6 antagonist (15/63, 24%), JAK inhibitors (13/63, 21%), CD20 inhibitor (11/63, 17%), and CTLA-4-Ig (5/63, 8%).

There were many chronological orders in which b/tsDMARDs were used (Supplementary Table S3). The most frequently used chronological order (*n* = 10) was a TNF inhibitor (first b/tsDMARD), followed by CTLA-4Ig (second b/tsDMARD), and then a JAK inhibitor (third b/tsDMARD). All other chronological orders of use accounted for 4% or less of all the possibilities.

After a patient becomes D2T RA, Jak inhibitors had a better median survival at 36 months compared to the other mechanisms of action when it was used as the third b/tsDMARD in the D2T RA group (Fig. 3). At their last treatment, 96% (*n* = 97/101) of D2T RA patients were still being treated with a b/tsDMARD (Table 1). More D2T RA patients than non-D2T RA patients were using chronic corticosteroids (D2T RA 16% versus non-D2T RA 5%, *p* = 0.0002) and slightly fewer were using csDMARDs (D2T RA 67% versus non-D2T RA 79%, *p* = 0.022).

## Discussion

Our study shows that in this cohort from an university hospital, D2T RA patients were more likely to have concomitant non-inflammatory pain and to discontinue medication due to intolerance when compared to b/tsDMARD good responders (non-D2T RA). Once a patient meets the D2T RA definition, JAK inhibitors had a longer survival than other b/tsDMARD mechanisms of action when used as the third b/tsDMARD. Fewer patients were managed with glucocorticoids at their most recent follow-up.

The reasons behind D2T RA are heterogeneous. Before switching or adding immunosuppressive medication, it may be useful to identify other causes of pain that can contribute to the patient’s symptoms [15]. The results of our study support the EULAR point to consider that non-inflammatory pain should be assessed in D2T RA patients before switching b/tsDMARDs. Other examples of comorbidities that may influence the clinical assessment of RA disease activity include osteoarthritis, damage from previous inflammation, obesity and depression [15]. The use of ultrasound may help distinguish active inflammatory disease from damage in D2T RA patients [4, 27, 28]. Strategies to manage these other causes of pain include setting realistic goals through shared-decision making, educating the patient on the multiple aetiologies of pain [15, 29] and including non-pharmacological interventions (e.g.: exercise, self-management interventions, psychological) [15]. Then, poorer drug tolerance is another reason for D2T RA [30]. Our D2T RA patients were more likely to discontinue their b/tsDMARD due to an intolerance than non-D2T RA patients. Although we didn’t find a difference in terms of intolerance due to infections or comorbidities, Takanashi et al. published on elderly Japanese patients with a higher comorbidity index [31]. These patients in Takanashi’s cohort were more likely to have D2T RA and infectious complications.

For the patient who has true multidrug resistance, JAK inhibitors may have more favorable outcomes when compared to other mechanisms of action for D2T RA patients [32–34]. The results from our study support their findings in that JAK inhibitors may be considered in D2T RA patients. The better outcomes on JAK inhibitors may be explained by the inhibition of a wider range of cytokines involved in the pathogenesis of RA and its analgesic effect [35, 36]. However, the widespread use of JAK inhibitors may be limited due to safety concerns [37–39]. Considering that some D2T RA patients have a higher comorbidity index [6, 31], only well-selected subgroups may benefit from this treatment option. Another therapeutic consideration is determining which patient needs chronic low-dose corticosteroids. In our cohort, the proportion of D2T RA patients on chronic corticosteroids was lower than in cohorts from other countries (16% versus from 29 to 74%) [5, 6, 18, 40]. We believe that steroid-sparing strategies may be considered even when a patient has a D2T RA. In our country, the use of chronic corticosteroids is less frequent than in other countries [24, 25], and this steroid-sparing treatment strategies may be facilitated by our universal healthcare coverage. It allows us to switch drugs when necessary, until the most effective b/tsDMARD is found.

## Comparison to other D2T RA cohorts

Comparing our cohort of D2T RA patients to other published cohorts (Table 2) needs to take into consideration the different inclusion criteria of our control group. In other studies, the non-D2T RA group included both csDMARD good responders and b/tsDMARD good responders, whereas our study only included b/tsDMARD good responders. The exclusion of csDMARD good responders can explain why this selected cohort has a high prevalence of D2T RA patients (24%).

**Table 2:** Comparison with other difficult-to-treat rheumatoid arthritis cohorts

	Our study (*n* = 101)	Roodenrijs 2021 (*n* = 52)	Takanashi 2021 (*n* = 173)	Ryu 2021 (*n* = 53)	Giollo 2022 (*n* = 48)	Jung 2023 (*n* = 271)	Hecquet 2023 (*n* = 76)	Novella-Navarro 2023 (*n* = 122)
Country	Canada	Netherlands	Japan	Japan	Italy	Korea	France	Spain
**Patient demographics**								
Age, years (S.D.)	63 (9)	60 (11)	66 (13)	57	–	54 (12)	59 (14)	61 (13)
Female, %	80	73	90	77	83	83	87	–
Age at onset, mean, years (S.D.)	49 (13)	42 (12)	51 (14)	52 (16)	46	–	–	43 (13)
Disease duration, years	16 (12^a^)	17 (9–25^b^)	15 (10^a^)	5 (2–14^b^)	2 (1–3^b^)	10 (5–15^b^)	15 (13^a^)	–
Time from diagnosis to first b/tsDMARD, years (Q1–Q3)	2 (1–8^b^)	6 (2–14^b^)	7 (9^a^)	–	–	–	–	5 (6)
RF positive, %	55	75	84	87	75	80	89	72
Anti-CCP positive, %	42	73	87	83	69	85	87	72
**Medication**								
b/tsDMARDs, median	4 (3–6^b^)	4 (3–6^b^)	2 (2^a^)	–	3 (3^a^)	2 (2^a^)	5 (1^a^)	–
b/tsDMARD mechanism of action, median	3 (3–4^b^)	3 (2–4^b^)	2 (1^a^)	–	–	–	–	–
csDMARD, %	67	71	61	77	44	74	55	–
b/tsDMARD, %	96	75	68	100	90	89	–	–
Glucocorticoid therapy, %	16	52	29	74	–	85	61	–
Prednisone dose, mg/day	–	8 (5–15^b^)	4 (2^a^)	4	5 (4–8^b^)	6 (3^a^)	5 (4^b^)	–
NSAIDs, %	27	40	–	–	27	–	–	–
**Potential factors contributing to D2T-RA**								
Adverse reaction on drug, %	39	94	–	–	–	36	–	–
Comorbidities leading to limited drug options, %	9	69	10	–	–	–	–	–
Concomitant non-inflammatory pain, %	28	38	–	–	2	–	–	20
Depression probable, %	13	15	2	–	6	–	–	29
Anxiety probable, %	9	17	–	–	6	–	–	29

*n* number of D2T RA patients, *b/tsDMARD* biologic or target synthetic disease-modifying antirheumatic drug, *csDMARD* conventional synthetic disease-modifying antirheumatic drug, *RF* rheumatoid factor, *anti-CCP* anti-cyclic citrullinated peptide, *ESR* erythrocyte sedimentation rate, *CRP* C-reactive protein, *NSAID* nonsteroidal anti-inflammatory drug

^a^ Standard deviation

^b^ Interquartile range (Q1–Q3)

This Canadian cohort had lower percentage of rheumatoid factor positive (51% versus from 75 to 87%) and anti-CCP positive (42% versus from 73 to 87%) patients compared to other cohort of D2T RA patients. Seronegativity may have clinical implications in difficult-to-treat RA. First, re-assessing the diagnosis may be important when a seronegative RA patient fails to respond to treatment, as other seronegative inflammatory arthritis can present similarly to RA and meet its classification criteria. Key features of these diseases may not appear over time when the patient is using a b/tsDMARD, which further complicates diagnosis. In our study, we excluded all patients with known concomitant rheumatic diseases that could cause chronic small joint polyarthritis (e.g.: systemic lupus erythematosus and RA overlap, skin psoriasis, inflammatory bowel disease). Also, some seronegative RA patients may have less inflammatory disease [41], and concomitant non-inflammatory pain can possibly explain their partial response to b/tsDMARD. Compared to other cohorts, we report a similar prevalence of concomitant non-inflammatory pain (28% versus 20–38%) in our D2T RA patients. Comorbidities had a similar impact on management in patients of our cohort compared to the cohort of Takanashi et al. (9% versus 10%), but less than the cohort of Roodenrijs et al. (9% versus 69%).

There are several limitations to our study. First, we were unable to retrospectively assess our patient’s socioeconomic status, coping strategies and expectations with regards to treatment. These factors may be important determinants in perceived treatment failure and may be covariates of non-inflammatory pain. Second, this study is subject to selection and information biases due to its retrospective and single-center nature. One challenge was to retrospectively apply the third EULAR criteria for D2T RA (management being perceived as problematic by the rheumatologist or patient), because there is subjectivity in the interpretation of the written medical records and patients were not contacted for this retrospective study [42, 43]. Third, we did not contact the patient’s pharmacy to adequately assess adherence. Fourth, a time-cohort bias could have given an advantage to RA treatments recently introduced, such as JAK inhibitors. A strength to our study is that we are the first North American cohort study using the EULAR D2T RA criteria. A second strength is that we are the first D2T RA study in our knowledge to use b/tsDMARD good responders as a control group. This gives us insight into factors that can make a RA patient difficult-to-treat after they are started on b/tsDMARDs.

Future studies could assess whether the use of ultrasound can help distinguish inflammatory disease activity from other causes of pain and if it can reduce possible unnecessary drug switch. Prospective study is also required to determine if one b/tsDMARD mechanism of action is superior to another in multidrug resistant RA.

## Conclusion

Compared to b/tsDMARD good responders, concomitant non-inflammatory pain seemed more prevalent in D2T RA patients from our cohort. Steroid-sparing strategies should be tried even in D2T RA patients. Future prospective research is needed to determine the predictive factors and the best therapeutic strategies to prevent a patient with RA from progressing to D2T RA, which may include the use of ultrasound to differentiate between active inflammatory activity and non-inflammatory pain. The role of JAK inhibitors versus other mechanisms of action in this group of D2T RA patients should also be prospectively explored.

## Electronic supplementary material

Below is the link to the electronic supplementary material.

Supplementary Material 1

## Data Availability

The datasets supporting the conclusions of this article are available from the corresponding author on reasonable request.
