# Supplementary material for: Characteristics of patients with difficult-to-treat rheumatoid arthritis: a descriptive retrospective cohort study
Source: Adv Rheumatol. 2024 Aug 6;64:55. doi: 10.1186/s42358-024-00396-6 (PMC13488725; doi:10.1186/s42358-024-00396-6)
Supplement: Supplementary file 1 [file 42358_2024_396_MOESM1_ESM.docx]

**Supplementary Material**

**Supplementary table S1**. Number of biologic or target synthetic disease-modifying antirheumatic drugs (b/tsDMARDs) used by difficult-to-treat rheumatoid arthritis (D2T-RA) patients

| b/tsDMARD position* | Number of patients | Classified as D2T RA (%) | not-yet D2T RA (%) |
| --- | --- | --- | --- |
| 1 | 101 | 0 | 101 (100) |
| 2 | 101 | 0 | 101 (100) |
| 3 | 101 | 59 (58) | 42 (42) |
| 4 | 70 | 63 (90) | 7 (10) |
| 5 | 49 | 48 (98) | 1 (2) |
| 6 | 28 | 29 (100) | 0 |
| 7 | 11 | 11 (100) | 0 |
| 8 | 6 | 6 (100) | 0 |

*chronological position in which the medication was used

**Supplementary** Table S2. Disease activity at the discontinuation of each b/tsDMARDs in D2T RA group

| b/tsDMARD position* | 1 | 2 | 3 | 4 | 5 | 6 | 7 |
| --- | --- | --- | --- | --- | --- | --- | --- |
| SJC (28), mean (S.D.) | 16 (7) | 16 (6) | 16 (6) | 14 (6) | 17 (6) | 17 (7) | 19 (10) |
| TJC (28), mean (S.D.) | 19 (10) | 21 (10) | 19 (9) | 17 (9) | 20 (9) | 19 (12) | 19 (10) |
| ESR, mean (S.D.) | 16 (18) | 16 (16) | 15 (15) | 11 (14) | 23 (26) | 16 (13) | 8 (5) |
| CRP, mean (S.D.) | 8 (16) | 11 (21) | 8 (13) | 6 (10) | 13 (17) | 7 (12) | 3 (3) |
| HAQ (S.D.) | 1.9 (0.5) | 1.9 (0.4) | 1.9 (0.5) | 1.8 (0.4) | 1.9 (0.5) | 1.6 (0.5) | 1.5 (0.5) |

*chronological position in which the medication was used

Legend

SJC: Swollen joint count (28); TJC: Tender joint count (28); ESR: erythrocyte sedimentation rate; CRP: C-reactive protein; HAQ: health assessment questionnaire.

**Supplementary table S3**. Most frequent chronological order of use of biologic or target synthetic disease-modifying antirheumatic drugs (b/tsDMARDs) in difficult-to-treat rheumatoid arthritis patients

| Position | 1^st^ agent | 2^nd^ agent | 3^rd^ agent | 4^th^ agent | Percentage |
| --- | --- | --- | --- | --- | --- |
| 1 | TNFi | CTLA-4Ig | Jaki | - | 10 |
| 2 | TNFi | TNFi | CTLA-4Ig | TNFi | 4 |
| 3 | TNFi | TNFi | CTLA-4Ig | IL-6 antagonist | 4 |
| 4 | TNFi | TNFi | CTLA-4Ig | - | 3 |
| 5 | CTLA-4Ig | IL-6 antagonist | Jaki | - | 3 |

TNFi: tumor necrosis factor inhibitor (adalimumab, etanercept, golimumab; certolizumab, infliximab); IL-6 antagonist: interleukin-6 antagonist (tocilizumab, sarilumab); CD20 inhibitor (rituximab); JAKi: janus kinase inhibitor (tofacitinib, baricitinib, upadacitinib), CTLA-4 Ig: cytotoxic T lymphocyte-associated antigen-4-immunoglobulin (abatacept)

**Supplementary Figure S1**. Duration on treatment of 1^st^ biologic or target synthetic disease-modifying antirheumatic drug (b/tsDMARD) in the difficult-to-treat rheumatoid arthritis (D2T RA) group up to 36 months

Legend

TNF inhibitor: tumor necrosis factor inhibitor (adalimumab, etanercept, golimumab; certolizumab, infliximab); JAK inhibitor: janus kinase inhibitor (tofacitinib, baricitinib, upadacitinib), CTLA-4 Ig: cytotoxic T lymphocyte-associated antigen-4-immunoglobulin (abatacept); Other mechanisms of action: IL-6 antagonist: interleukin-6 antagonist (tocilizumab, sarilumab), CD20 inhibitor (rituximab)

**Supplementary Figure S2**. Duration on treatment of 2^nd^ biologic or target synthetic disease-modifying antirheumatic drug (b/tsDMARD) in the difficult-to-treat rheumatoid arthritis (D2T RA) group up to 36 months

Legend

TNF inhibitor: tumor necrosis factor inhibitor (adalimumab, etanercept, golimumab; certolizumab, infliximab); JAK inhibitor: janus kinase inhibitor (tofacitinib, baricitinib, upadacitinib), CTLA-4 Ig: cytotoxic T lymphocyte-associated antigen-4-immunoglobulin (abatacept); Other mechanisms of action: IL-6 antagonist: interleukin-6 antagonist (tocilizumab, sarilumab), CD20 inhibitor (rituximab)

**Supplementary Figure S3**. Duration on treatment of 1^st^ biologic or target synthetic disease-modifying antirheumatic drug (b/tsDMARD) in the non-difficult-to-treat rheumatoid arthritis (non-D2T RA) group up to 36 months

Legend

TNF inhibitor: tumor necrosis factor inhibitor (adalimumab, etanercept, golimumab; certolizumab, infliximab); JAK inhibitor: janus kinase inhibitor (tofacitinib, baricitinib, upadacitinib), CTLA-4 Ig: cytotoxic T lymphocyte-associated antigen-4-immunoglobulin (abatacept); Other mechanisms of action: IL-6 antagonist: interleukin-6 antagonist (tocilizumab, sarilumab), CD20 inhibitor (rituximab)

**Supplementary Figure S5**. Duration on treatment of 2^nd^ biologic or target synthetic disease-modifying antirheumatic drug (b/tsDMARD) in the non-difficult-to-treat rheumatoid arthritis (non-D2T RA) group up to 36 months

Legend

TNF inhibitor: tumor necrosis factor inhibitor (adalimumab, etanercept, golimumab; certolizumab, infliximab); JAK inhibitor: janus kinase inhibitor (tofacitinib, baricitinib, upadacitinib), CTLA-4 Ig: cytotoxic T lymphocyte-associated antigen-4-immunoglobulin (abatacept); Other mechanisms of action: IL-6 antagonist: interleukin-6 antagonist (tocilizumab, sarilumab), CD20 inhibitor (rituximab)
